# Development of Privacy Features on Anecdata.org, a Free Citizen Science Platform for Collecting Datasets for Climate Change and Related Projects

**DOI:** 10.3389/fclim.2021.620100

**Published:** 2021-04-30

**Authors:** Cait Bailey, Anna Farrell, Turam Purty, Ashley Taylor, Jane Disney

**Affiliations:** Mount Desert Island Biological Laboratory, Bar Harbor, ME, United States

**Keywords:** citizen science, data privacy, geoprivacy, anonymity, data to action

## Abstract

The Anecdata website and its corresponding mobile app provide unique features to meet the needs of a wide variety of diverse citizen science projects from across the world. The platform has been developed with the help of continuous feedback from community partners, project leaders, and website users and currently hosts more than 200 projects. Over 8,000 registered users have contributed more than 30,000 images and over 50,000 observations since the platform became open to the public in 2014. From its inception, one of the core tenets of Anecdata’s mission has been to make data from citizen science projects freely accessible to project participants and the general public, and in the platform’s first few years, it followed a completely open data access model. As the platform has grown, hosting ever more projects, we have found that this model does not meet all project needs, especially where endangered species, property access rights, participant safety in the field, and personal privacy are concerned. We first introduced features for data and user privacy as part of “All About Arsenic,” a National Institutes of Health (NIH)/National Institute of General Medical Sciences (NIGMS) Science Education Partnership Award (SEPA)-funded project at MDI Biological Laboratory, which engages middle and high school teachers and students from schools across Maine and New Hampshire in sampling their home well water for analysis of arsenic and other heavy metals. In order to host this project on Anecdata, we developed features for spatial privacy or “geoprivacy” to conceal the coordinates of samplers’ homes, partial data redaction tools we call “private fields” to withhold certain sample registration questions from public datasets, and “participant anonymity” to conceal which user account uploaded an observation. We describe the impetus for the creation of these features, challenges we encountered, and our technical approach. While these features were originally developed for the purposes of a public health and science literacy project, they are now available to all project leaders setting up projects on Anecdata.org and have been adopted by a number of projects, including Mass Audubon’s Eastern Meadowlark Survey, South Carolina Aquarium’s SeaRise, and Coastal Signs of the Seasons (SOS) Monitoring projects.

## INTRODUCTION

### Citizen Science and Evolution of Anecdata

Citizen science, or the involvement of citizens in scientific research, is an effective strategy for expanding capacity for science and fostering the use of science in decision-making about complex problems (Wals et al., 2014; Dillon et al., 2016). Anecdata.org is an online platform developed at the MDI Biological Laboratory’s Community Environmental Health Lab for the collection of observational data from citizen scientists that is uniquely designed to enable project leaders and participants to utilize their data to enact change (Disney et al., 2017).

The development of Anecdata started in 2014 to provide a data management system for several citizen science projects run by the Community Environmental Health Lab, ranging from bay monitoring to seagrass restoration. Until then, the projects used a combination of Microsoft Excel sheets and Access databases to store data, which became very prone to errors as projects scaled up and made it difficult for the administrative and research team to effectively share data with collaborators and community members in a timely fashion.

Over the years, Anecdata evolved as an online platform for citizen science data collection, aggregation, and analysis through continuous feedback and suggestions from community partners who reached out to our team to host their projects. The development of the platform has followed an Agile software development methodology as defined in the Agile Manifesto, where features are developed by prioritizing and valuing “individuals and interactions over process and tools, working software over comprehensive documentation, customer collaboration over contract negotiations, and responding to change over following a plan” (Hazzan and Dubinsky, 2014).

Today, Anecdata is freely available to citizen groups and community partners around the world. As of publication, it is home to more than 200 projects, where more than 8,000 registered users have contributed over 30,000 images and more than 50,000 observations. Anecdata also continues to serve as a key platform for projects at the Community Environmental Health Lab, especially “All About Arsenic,” a 5-year National Institutes of Health (NIH)/National Institute of General Medical Sciences (NIGMS) Science Education Partnership Award (SEPA)-funded project that focuses on building data literacy among middle and high school students while engaging them in sampling their home well water for arsenic and other contaminants and sharing their findings within their local and regional communities. This is the project that provided the impetus for the development of data privacy features on Anecdata.

### Privacy Features on Anecdata

Managing a large repository of online citizen science datasets opens many avenues for developing best practices for citizen science digital data management, including ensuring privacy of certain data types. A high-level overview of data management in citizen science includes individual research topics such as data acquisition, data quality, data infrastructure, data security, data governance, data documentation, data access, data services, and data integration (Bowser et al., 2020).

Many projects on Anecdata have informed the development of new functionalities on the platform. “All About Arsenic”^1^ is the first project where we systematically developed three such features: (1) “geoprivacy,” so that sample site coordinates could be obscured; (2) “private fields,” so that certain data fields could be concealed from public view; and (3) “participant anonymity,” so that the identity of the person who originally registered a sample is not revealed. These features, defined in Table 1, subsequently became available for all projects on Anecdata. Although most data are available for the public to view and download, fields that have been marked as private are only available to project administrators.

Privacy features are critical components of many citizen science projects where protecting the privacy and security of individual participants is essential. Incorporating these features in the design and development of the citizen science platform allows project leaders to support their project participants in making informed and safe decisions about their personally identifiable information (Bowser et al., 2014).

There are multiple reasons why “All About Arsenic” project participants want their personal information obscured. The potential health impacts of arsenic exposure raise issues of medical privacy. In addition, high levels of arsenic in well water could affect property values. A study on the effect of elevated arsenic levels in well water on home values in two Maine towns showed no significant negative impact after 2 years (Boyle et al., 2010). However, a later survey of private well owners in Maine revealed the belief that mitigating arsenic in well water would increase the value of their homes (Flanagan et al., 2015). The relationship between well water quality and negative impact on home values has been documented in other parts of the nation as well (Guignet et al., 2016).

The new privacy features stemming from the “All About Arsenic” project are now available and accessible to all projects on Anecdata and provide vital functionality for groups that are crowdsourcing a wide variety of information that requires data privacy. For these projects, datasets (which are considered privileged) can be downloaded by project administrators but not the general public.

One of the first projects to adopt new data privacy features after they were introduced on the Anecdata site was Coastal Signs of the Seasons (SOS) Monitoring. This program is an offshoot of a New England-wide phenology program that engages citizen scientists in observing 19 upland and coastal indicator species with two main objectives. The first is to characterize the biological effects of climate change through the collection of phenology data and the second is to empower citizens to become a part of the solution to climate change by participating in research comparing the current timing of life cycle events for individual species with historically documented events such as leaf-out, flowering, and gamete production (Stancioff et al., 2017). Other climate change and related projects on Anecdata soon followed suit and adopted privacy features.

While individual projects may have policies that adhere to laws and ethical standards (Guerrini et al., 2018), technology platforms such as Anecdata have a role to play in promoting ethics in citizen science by building in features that provide options and support for privacy controls at both the individual and project levels (Bowser et al., 2014).

As we enter an era where citizen science and open science receive greater recognition, we can celebrate that information is more freely available to everyone for use in advocacy, to promote environmental improvements, to enhance human health, to protect wildlife, and more. At the same time, there are concerns about data quality, stewardship, privacy, security, and control (Bowser et al., 2020), particularly in the case of data that relate to human health (Majumder and McGuire, 2020). Anecdata is in the company of several citizen science platforms that have aimed to achieve a balance between unrestricted public access to data and levels of privacy for project leaders and data contributors, such as CitSci.org (Wang et al., 2015; Lynn et al., 2019), Open Humans (Tzovaras et al., 2019), and iNaturalist (Bowser et al., 2014).

Anecdata supports location and user privacy features and provides the option for any additional data fields to be kept private. In this paper, we present our “All About Arsenic” project as a case study in data privacy and relate it to an early adopter of data privacy features on Anecdata.org, SOS Monitoring.

## METHODS

### Anecdata Technology Stack Description

Anecdata is an online platform composed of a server-side data management system, a public Web interface, and mobile apps for iOS and Android. Anecdata was designed to manage and publicly share our project data at the Community Environmental Health Lab. It is freely available for others to use for projects that serve the public good. While originally envisioned for use with environmental and conservation data, it is now being used by project leaders and participants to collect and share a range of dataset types, including public health, and city planning.

Both the website and the mobile app exchange data with the Anecdata server using the same application programming interface (API) endpoints, which send and receive structured data such as lists of observations, chat messages, or user profiles. The Anecdata server is written in PHP using the CakePHP framework and uses the MariaDB relational database for data storage. The Anecdata website and mobile app are both developed in TypeScript using the Angular framework. The mobile app additionally uses the Ionic framework to provide a native user experience and interface with the device’s hardware. By using Angular across all platforms (both mobile and website), the shared code reduces the overall development time when introducing new features. All features developed for one project can be easily replicated across and made available to all projects on the platform.

### Data Collection Schema on Anecdata

**FIGURE 1 |:**
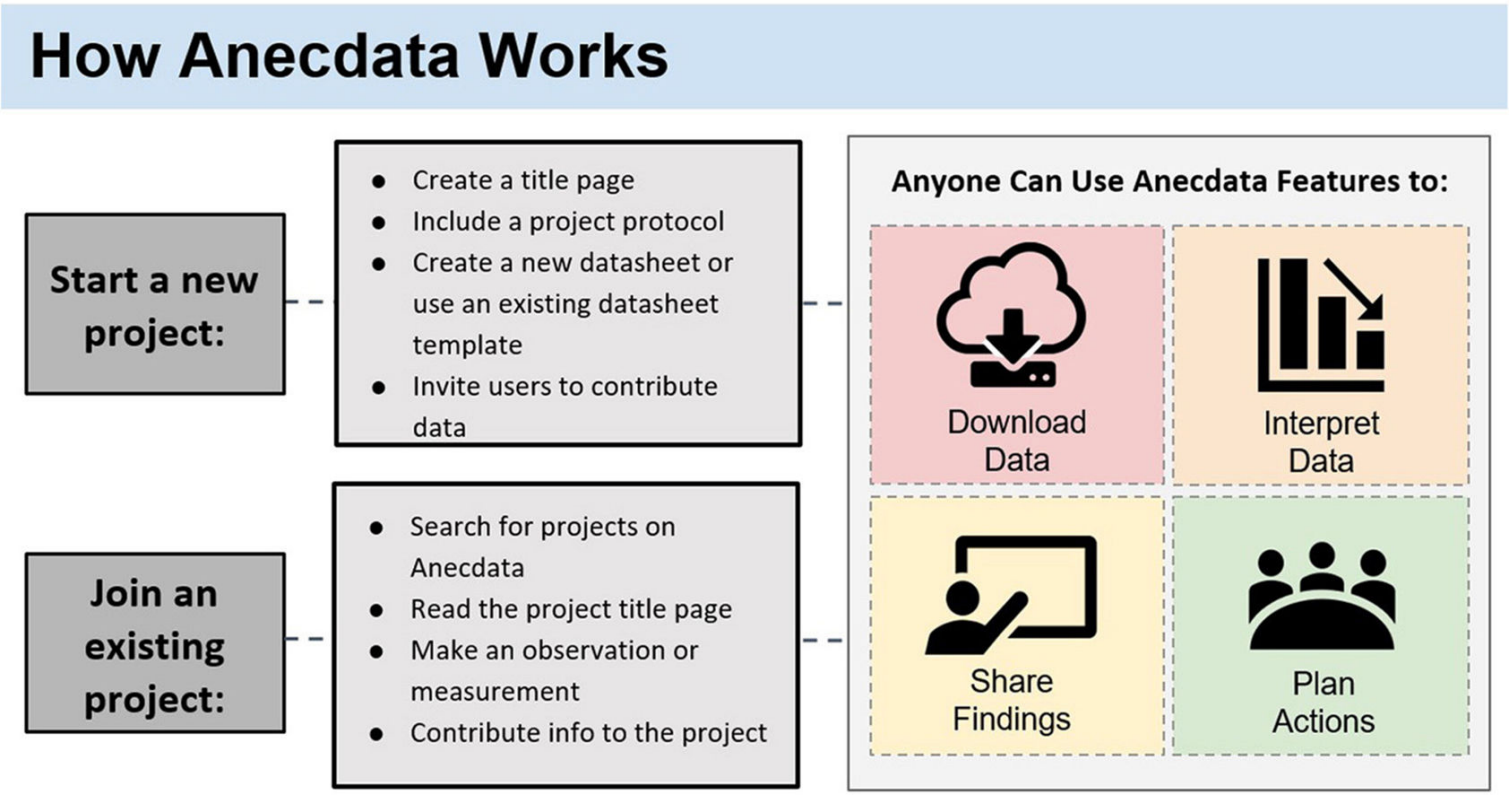
How Anecdata works. Anecdata is a citizen science platform that welcomes new projects, some of which are open to new participants joining. Anyone can download non-private data for analysis and interpretation, share the data with others, and use the data to plan actions aimed at effecting change at any societal level.

The sequence of steps for setting up a new citizen science project or getting involved in an existing project on Anecdata is depicted in Figure 1. For everyone, the first step involves creating a user account with an email address and password. A date of birth column is captured during user account registration to ensure that all users are above the age of 13, as US federal law requires that anyone using online platforms collecting personally identifiable information be at least 13 years of age.

Projects, in the context of Anecdata, are pages that have been created by one or more project administrators with the purpose of gathering observations to fill a data need. Data are shared with these projects by participants in the form of observations.

**FIGURE 2 |:**
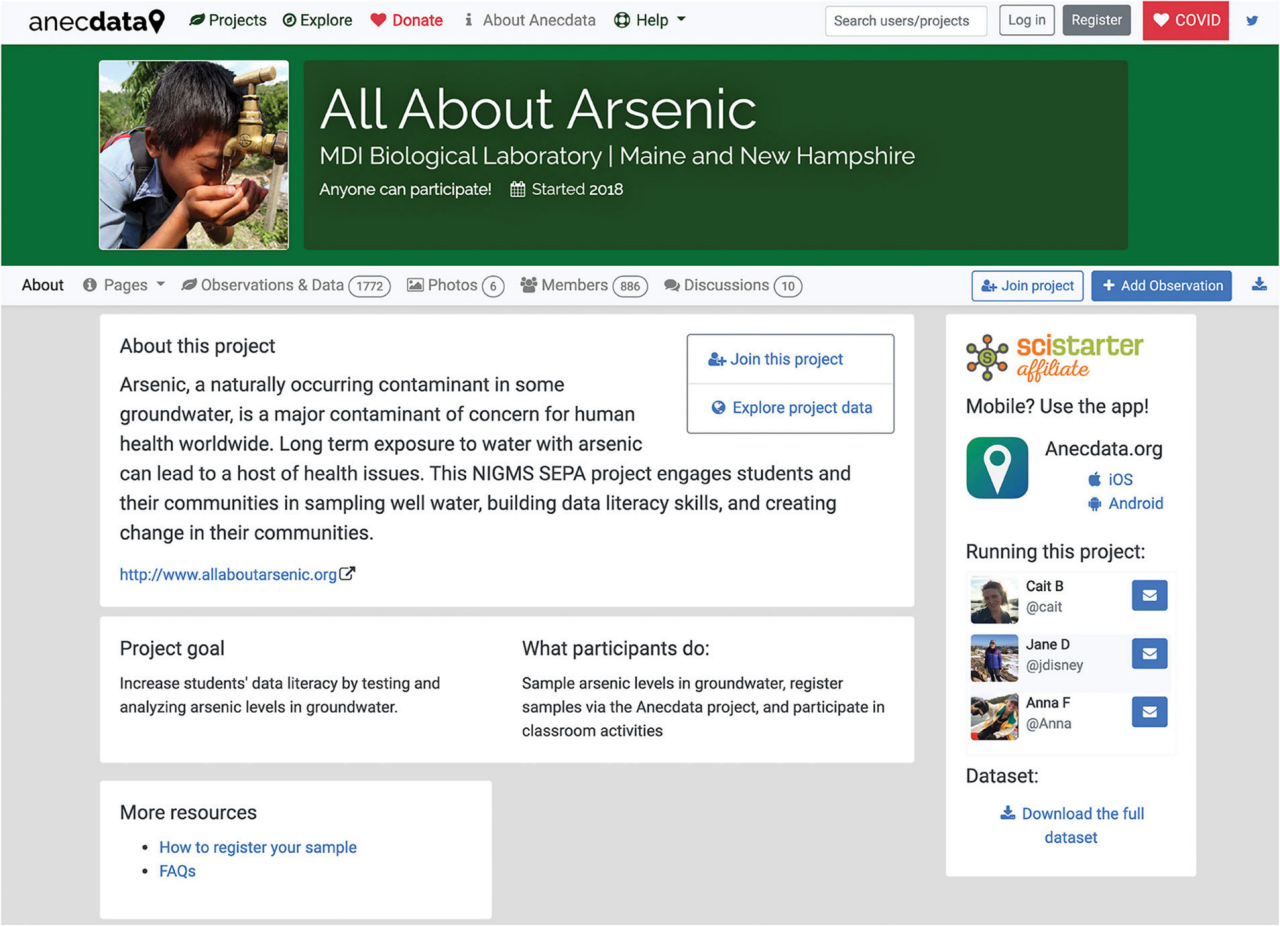
A typical project page on Anecdata describes the project and the project goals and provides instructions to participants. In the case of “All About Arsenic,” more details about the project are provided in a link to the project website.

Project administrators use the project designer tool to enter information about their project’s goals, protocols, and other essential information for project participants. This generates a custom project page from an established project page template (Figure 2). The data schema of a project can be customized to suit the needs of the project using the “datasheet” designer tool.

The “datasheet” designer tool provides an interface for creating a list (rows and columns) of named datasheet fields that participants use to enter data and offers multiple base data inputs that project administrators can choose, including text inputs, numbers, yes/no checkboxes, controlled-vocabulary dropdown menus, date, time, and geospatial coordinates. The datasheet designer also offers templates for common use cases such as litter cleanups, animal observations, water quality monitoring, and collecting biological specimens in the field.

The structure of the Anecdata datasheet system allows for the entry of two categories of data:

1. **Parent fields**, which are fields that pertain directly to all data on the datasheet. In the case of the “All About Arsenic” project, examples of these fields include the name of the student’s school, the name of the legal guardian, and well type.
2. **Child fields**, which are repeating blocks of questions that allow the participant to log multiple entries. In the “All about Arsenic” project, students may submit multiple water samples (pre- and post-filtration, or from different locations in the house, such as the kitchen sink and outside faucet), and the child fields on the datasheet pertain to an individual water sample. Examples of these fields include the sample vial ID number, where the sample was collected in the home, the type of filtration system used (if any), and additional comments.

Every time participants visit an Anecdata project page and begin a new observation, they are presented with a blank datasheet with the data fields that project administrators have designed. After all data have been entered and saved, the observation becomes publicly visible on the Anecdata website.

The “All About Arsenic” project workflow provides project participants with the option to share their private data with a state agency; in Maine, well water analysis and associated metadata are shared with the Maine Center for Disease Control (CDC), and in New Hampshire, they are shared with the New Hampshire Department of Environmental Services (DES). Before entering any data into the “All About Arsenic” data form on Anecdata, the project participants encounter a disclosure question that requires them to provide or deny permission for the sharing of their private data (exact latitude and longitude, parent and student first name and last name, and home address).

Participants fill out the datasheet to register the spatial coordinates of where their sample was collected, indicate whether the sample was filtered, and share other related metadata (Table 2). The well water samples are brought to school and then shipped by teachers to the Community Environmental Health Lab where the labels on each tube are cross-checked with teacher log sheets and sample registrations on Anecdata. Cross-checked batches of well water samples from one or more classrooms are sent to the Trace Element Analysis Core (TEAC) at Dartmouth College for analysis of 14 variables including antimony, arsenic, barium, beryllium, cadmium, chromium, copper, iron, lead, manganese, nickel, selenium, thallium, and uranium.

Datasets for each batch are returned to the Community Environmental Health Lab from TEAC in Excel file format. Using a unique uploader feature on Anecdata, the analytic results are aligned with the metadata in the “All About Arsenic” project. Teachers alert students when sample results are ready for viewing. Parents and students use a sample lookup tool on the “All About Arsenic” project website^2^ to retrieve their well water test results. When they enter their sample number, a pop-up display informs them of whether their sample data are available or not; if results are available, the user is automatically redirected to the Anecdata observation page for their well water test results. We added a data validation feature to the “All About Arsenic” project, which displays the maximum contaminant level (MCL) for each analyte next to the result, highlighting samples that are below the EPA MCL in green and those that are at or above the MCL in red. The complete dataset for each sample can be downloaded as a PDF so that each family has a record of its individual water sample results and associated metadata. These customized features were developed for the “All About Arsenic” project and are now available options for other related projects on Anecdata.

### Development of Data Privacy Features on Anecdata

From a data management and privacy standpoint, the implementation of the “All About Arsenic” project posed several challenges because at the time, Anecdata had an open model whereby all observations were visibly linked to the participant who shared them. We recognized that for the purposes of this project, we needed to protect the locations of participants’ homes as well as make sure the identities of sample registrants were protected. We developed a way to obscure this information while retaining the ID of the original observer, so they can update their sample registration later if needed.

We addressed the issue of participant privacy by obscuring the account that registered a sample and questions on the sample registration that would require personally identifiable information. By obscuring this information, effectively making it inaccessible upon public download, we anticipated that more individuals would feel comfortable about participating in the “All About Arsenic” project or other projects with similar data privacy needs.

### Development of the Anonymity Feature

**FIGURE 3 |:**
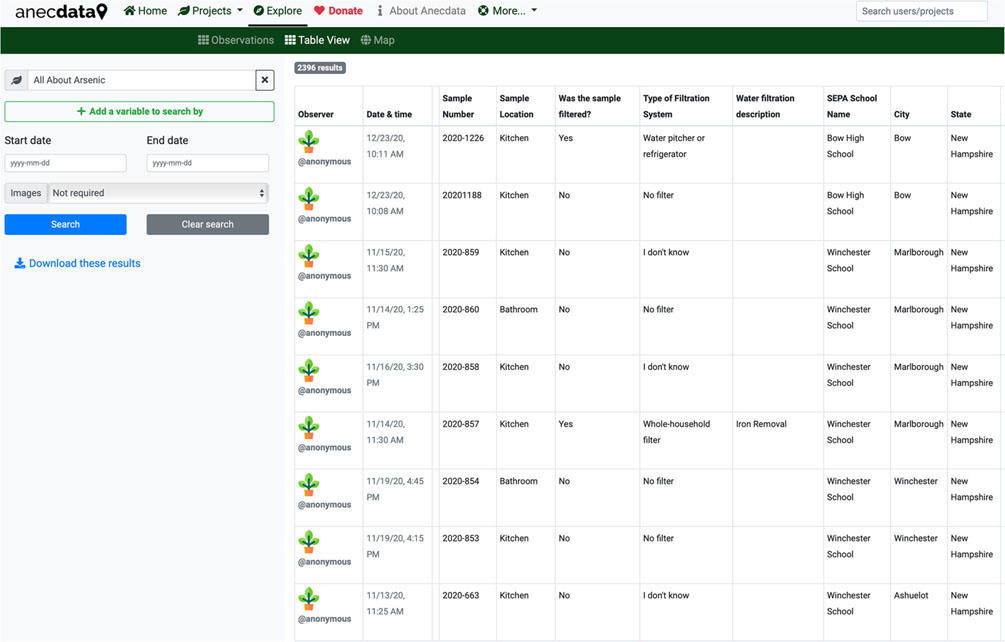
This screenshot of participant information on Anecdata shows that each participant is identified as @anonymous.

In order to make observations anonymous, the first step was to add a Boolean variable to the project’s settings, called **anonymize**, which defaults to **false** in all projects unless otherwise selected by a project administrator. The Anecdata software checks this variable when saving new observations:

1. When **anonymize** is **false**, it stores the ID of the currently logged-in user with the observation data as it normally would.
2. When **anonymize** is **true**, **a special account called @Anonymous** is displayed as the creator of the observation (Figure 3). We also add a record to a table with two values, **post_id** (the ID of the observation) and **user_id** (the ID of the currently logged-in user). Data from this table are never displayed directly from any of Anecdata’s API endpoints.

When retrieving an observation from the Anecdata API, we set an additional **edit** variable in the payload returned by the server that informs the user interface whether to display an Edit button that the user can use to correct any mistakes they may have made. For every observation displayed to the user, the Anecdata server-side software checks multiple conditions and sets edit accordingly (Table 3).

The benefit of this approach is that instead of needing to filter observations every time they are read from the database to ensure that the link to the originating user’s account is removed, we simply never store the link at all in the standard table of observations and only refer to the original table when we need to check access permissions for the purposes of editing an observation.

### Development of Spatial Privacy Feature

Our approach to spatial privacy, also known as geoprivacy, is similar to the spatial privacy model used by iNaturalist for the protection of endangered species (iNaturalist, 2019). While the exact coordinates of observations are available to project administrators, the publicly available coordinates are obscured by adding a random floating-point number between -0.1 and 0.1 to the latitude and longitude (Table 4). This random number is stored when the observation is saved and not generated each time the observation is read from the database, thereby preventing users from guessing where an observation is by refreshing the page repeatedly to deduce the exact coordinates.

The first step in implementing this feature was adding a new Boolean switch on projects, **geoprivacy**, which defaults to false unless otherwise chosen by a project administrator.

All Anecdata observations are located spatially using **lat** and **lng** decimal columns to store latitude and longitude in the database. We added two new columns, **private**_lat and **private**_lng, to store the exact unobscured coordinates of every observation.

**FIGURE 4 |:**
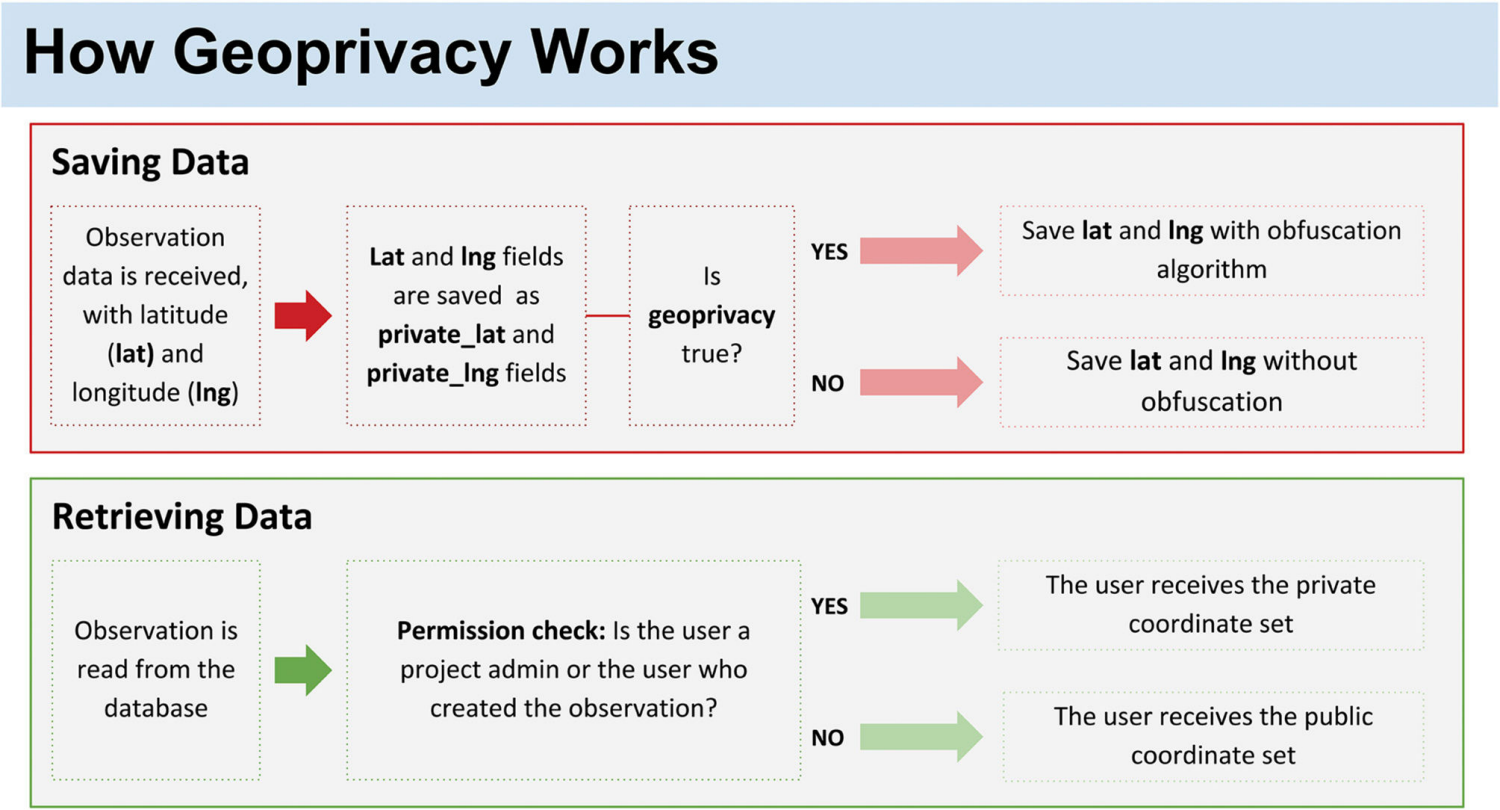
How geoprivacy works. When geoprivacy is needed for a project, the lat and lng are saved with an obfuscation algorithm. When data are retrieved with geoprivacy options in place, there is a permission check to ensure that privacy is maintained.

We then added a function to the Anecdata server-side software that checks when saving an observation whether the corresponding project’s geoprivacy is true or false (Figure 4).

**FIGURE 5 |:**
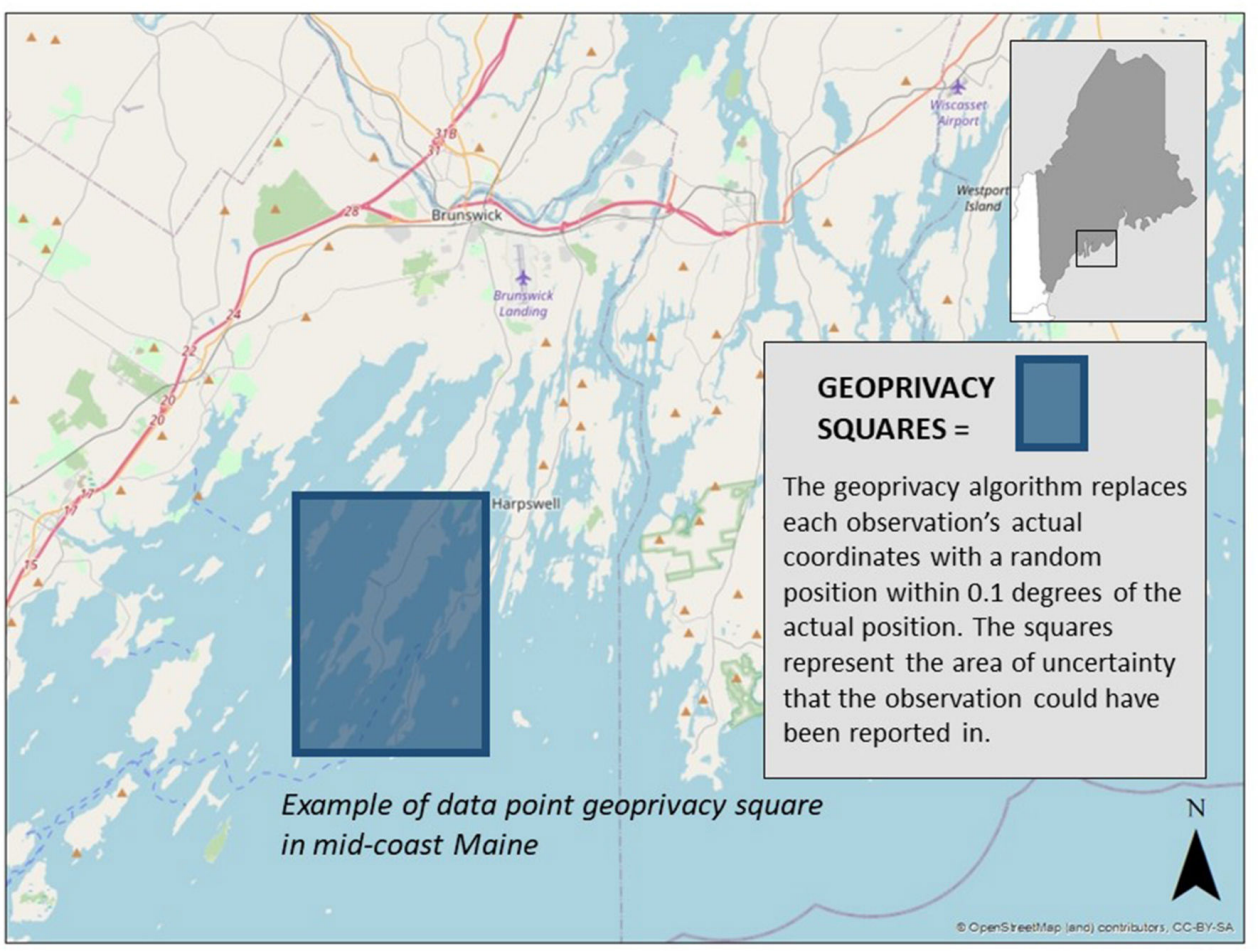
Geoprivacy squares. All well water variables are associated with obfuscated lat/long measurements that put the data point somewhere within each square, not close enough to the actual location to reveal the address or identity of the project participant.

The result of this is that all observations in the project have publicly displayed latitudes and longitudes that are (+/–) 0.1 degrees away from their actual location. These can be thought of as “boxes of uncertainty” on a map (Figure 5).

### Development of the Private Fields Feature

A key privacy concern in the sample registration process for the “All About Arsenic” project is protecting the identities of participants. We needed to collect the names and home addresses of participants and keep these data private while keeping other aspects of their sample registrations, such as sample number, well type, and sampling date, public.

To implement this, we added an additional column to our table of datasheet template fields called private. In order to prevent a data breach, fields that have been marked as private are not saved to the standard fields table that all other data are saved in, but rather a separate table that is not normally accessed while viewing and analyzing data.

When a user or project administrator edits an observation or when a project administrator downloads a privileged dataset, the Anecdata software checks the user’s privileges before running a separate data query that loads all the private fields from a separate table and displays them on the data entry form or in the export CSV file as if it were any other column. This approach is similar to the one we use for anonymity; instead of marking data as private and actively removing them every time observations are accessed, we store it in a separate table and only include them when the data endpoint explicitly needs it and we have ascertained that the user has access privileges.

After privacy features were developed and available to all project administrators on Anecdata, numerous projects began to adopt these features. In order to understand how these features were helpful, we asked project leaders for feedback. A feedback survey was emailed to all project administrators who had signed up to receive updates from our team. The feedback survey was sent via email to project administrators in line with Agile principles for providing a sustainable means for the users of privacy features to reflect on how they could be made more effective and efficient (Hazzan and Dubinsky, 2014). The following three questions were asked of 200 project administrators:

1. Can you comment on how privacy features such as geoprivacy and/or anonymity have been helpful in your work?
2. How satisfied are you with the current privacy features on a scale of 1–5? (1, low-5, high)
3. What can we do to improve the privacy features on Anecdata?

## RESULTS

While we designed privacy features with our “All About Arsenic” project’s needs in mind, many other projects on Anecdata are now using these same features. Since privacy features were introduced with the “All About Arsenic” project in 2018, 22 additional projects have begun using one or more privacy features (Table 5). Of these projects, 10 are using private fields, 15 are using geoprivacy, and five are using the participant anonymity features.

Climate change-related projects using private fields include “MaMA (Monitoring and Managing Ash) Monitoring Plots Network”^3^ in which participants monitor ash trees on an annual basis to determine mortality due to the invasive insect, emerald ash borer, and “Great Green Crab Hunt,”^4^ which involves monitoring coastal New England habitats for the invasive green crabs. Projects using geoprivacy to obscure the exact coordinates of observations include Mass Audubon’s “Eastern Meadowlark Survey,”^5^ which collects observations of meadowlark presence and absence at 434 sites across Massachusetts, and the University of Maine’s “Coastal SOS Monitoring” project,^6^ which collects phenology data on rockweed as an important climate change indicator along the coast of Maine.

We note that participant anonymity is not used as frequently as other privacy features, accounting for only five of the 23 projects on Anecdata that are utilizing these features. Three of the five are school-based such as our “All About Arsenic”^7^ project, which engages secondary school students in collecting private well water samples for analysis of arsenic and other contaminants, the “Dartmouth Dragonfly Mercury Project,”^8^ which involves students in collecting dragonfly larvae from streams for mercury analysis, and “NASA’s Lower the Boom” project,^9^ which enlists high school students in collecting measurements of background noise samples to determine how quiet supersonic jetliners would have to be in order to not cause a disturbance when flying across the continental US. Without the anonymity feature, locations could be deduced even with the geospatial privacy feature in place, such as Mass Audubon’s “Barn & Cliff Swallow Nesting Sites” project,^10^ which asks local birders to identify farm buildings and other structures that may be used by nesting swallows. Given that some project participants might identify their own farm buildings, participant anonymity is as necessary as geoprivacy in order to protect the location of these nesting sites.

### Feedback on Privacy Features

We requested feedback from over 200 project administrators, 22 of whom (aside from our own “All About Arsenic” project) are currently using privacy features. We wanted to know how helpful the features were, the level of satisfaction with the features, and suggestions for improving the features. We received feedback from 11 project administrators over a 2-week period. Based on our analysis of project leader feedback on privacy features, we learned that these features are useful for reasons that we did not necessarily anticipate. We also learned about barriers and challenges for Anecdata users. Of the 11 respondents, seven currently use privacy features, two do not use the features because they are too restrictive, one does not use the features for reasons that were not stated, and one has intentions to use features in the future. One respondent commented, “I think the fact that you are asking is pretty stellar.”

**FIGURE 6 |:**
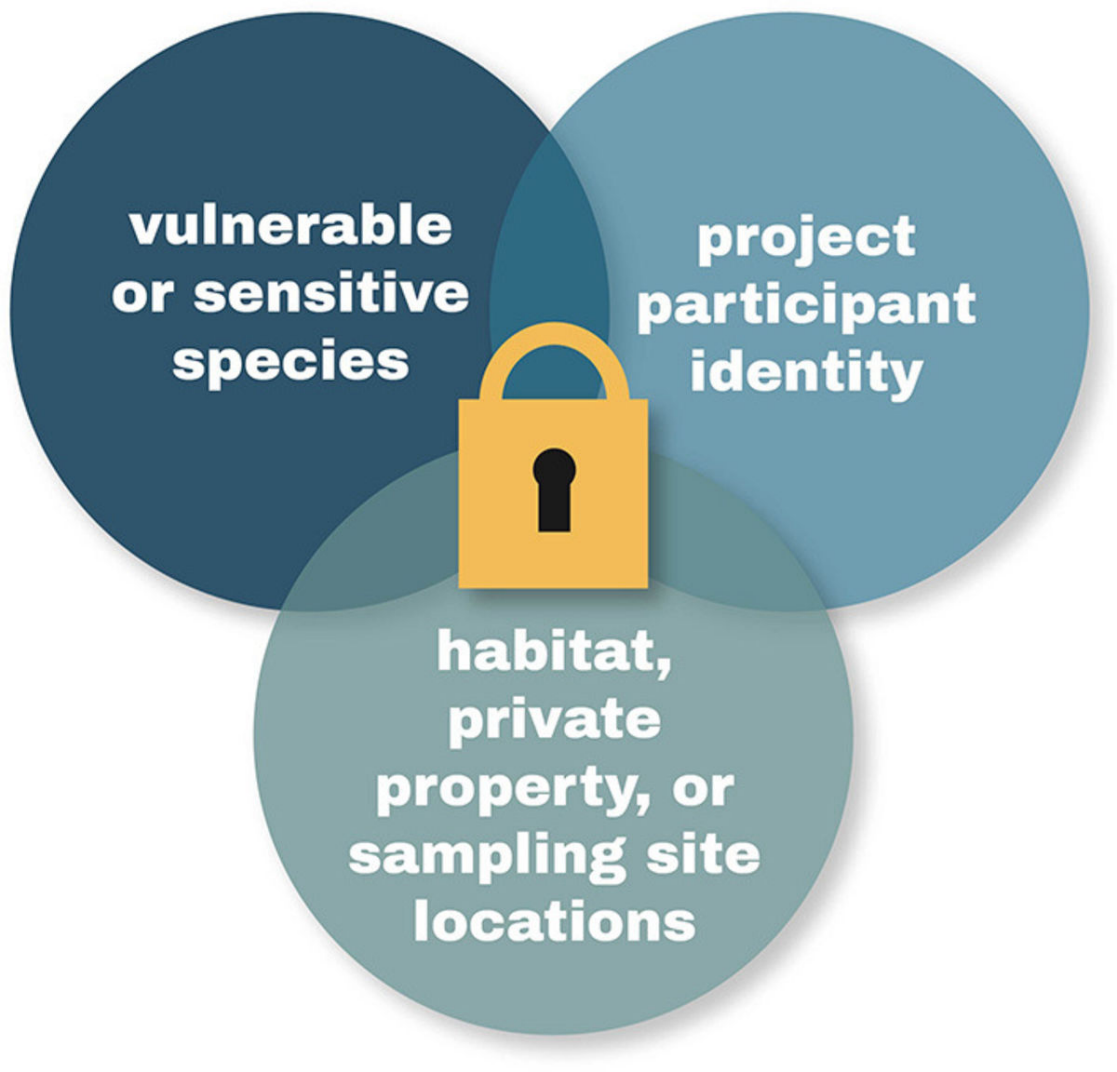
Themes emerging from project administrator feedback. Expressed needs for data privacy included private fields for species data, geoprivacy for private property and sample site locations, and anonymity for project participants, in particular, schoolchildren.

Three themes emerged from the feedback. These themes relate to protection of endangered species and their habitats, privacy of students involved in school-based projects and other project participants, and maintenance of property owner privacy and rights. Figure 6 depicts how a suite of complementary privacy features can help to address multiple concerns across multiple projects with common reasons for wanting to preserve data privacy.

### Flexibility for Privacy Settings

We learned from project administrator feedback that more flexibility is needed in privacy controls. Several project leaders indicated that they would like a higher level of control in project settings that allow them to set the degree to which location data are obscured:

“For our measurements, it would be good if indeed you wouldn’t see the actual house or garden where a measurement was taken but the current rounding of the GPS coordinates is too much. If it would be possible to choose a certain level of geoprivacy and the coordinates could for example also be rounded to two decimals that would be better.”

“We use Anecdata for our precipitation measurements... However, we realized that there could be some privacy issues. Activating the geoprivacy feature doesn’t help in our case, since precipitation can change over small spatial units. Long story [short], it would be very handy to have the option of geoprivacy with different rounding options.”

In addition, a respondent suggested using avatars or nicknames instead of names as an alternative to having “anonymous” as the default designation in the participant privacy feature. This could also be useful if only some people need or would want to have their names obscured on the project page.

### Communicating Data Privacy Features to Project Participants

A conversation with the “Coastal SOS Monitoring” project administrator informed us of the process used to make property owners and data collectors aware of the importance of data privacy, especially as it relates to location of sampling sites on private shorelines. Project leaders or participants inform property owners that their site location is not shared and that no one can access the participant data portal without permission from a project coordinator. This gives many coastal property owners a sense of security that their site location will be obscured and kept confidential by the Anecdata system. One concern for coastal private property owners who give permission for volunteers to access the shoreline adjacent to their property is that other people will then view their property as open and accessible to the general public. Information about data privacy is provided to participants in both their in-person and online trainings. While “Coastal SOS Monitoring” project data are shared with scientists studying climate change as it relates to coastal ecology, site locations are not revealed. Privacy features can address different kinds of issues that come up related to private property. Based on feedback from project leaders using the Anecdata platform, it is clear that a formal usability study on privacy across this broad range of projects will help us to better understand why data privacy features are being used and how they can meet the growing needs for data privacy by various citizen science projects.

### Technological Solutions to Human Errors

Early in the “All About Arsenic” project, non-obfuscated latitude and longitude data were inadvertently uploaded to our private arsenic platform on the Tuva data literacy website. During the time the actual coordinate data were accessible, it would have been possible for a student or other project member to use the mapping feature on the Tuva website and determine the well water quality status at points on named streets and possibly deduce the homeowner’s identity. However, since there are no property lines on Tuva maps, it was unlikely that points could be correlated with individual households. Nonetheless, this made it clear to us that we needed to address this potential for error.

In order to address this, we updated the standard CSV downloader used by all projects on Anecdata to include a toggle switch for administrators that lets them switch between downloading their publicly available dataset and their privileged dataset. In order to help prevent the inadvertent sharing of datasets after they have been downloaded, privileged dataset downloads have their filenames prefixed with “**admin**” and the headers of all private columns are prefixed with “**private**”.

## DISCUSSION

We recognize the role that technical platforms play in ensuring that citizen science projects are undertaken in responsible and ethical fashions that ensure privacy and/or anonymity of participants, permissions by participants for disclosure of data in private fields, and location privacy where necessary. When these features are made available, then project leaders setting up projects on these platforms can be guided toward more ethical projects by virtue of these available options.

“All About Arsenic” is an example of how metadata privacy can be achieved in an otherwise public-facing project. By combining geospatial, anonymity, and private field features in this project, with an option for providing permission for full disclosure of all project data, we have made it possible for this emerging citizen science dataset to affect change at the community level, protect public health, and inform public health policy. We have anecdotal reports of families installing well water filtration systems to deal with high arsenic levels in their drinking water. We are planning a follow-up study with all participants to determine actions taken in response to receiving well water test results and receiving informational materials and/or attending community outreach events hosted by students involved in the project.

**FIGURE 7 |:**
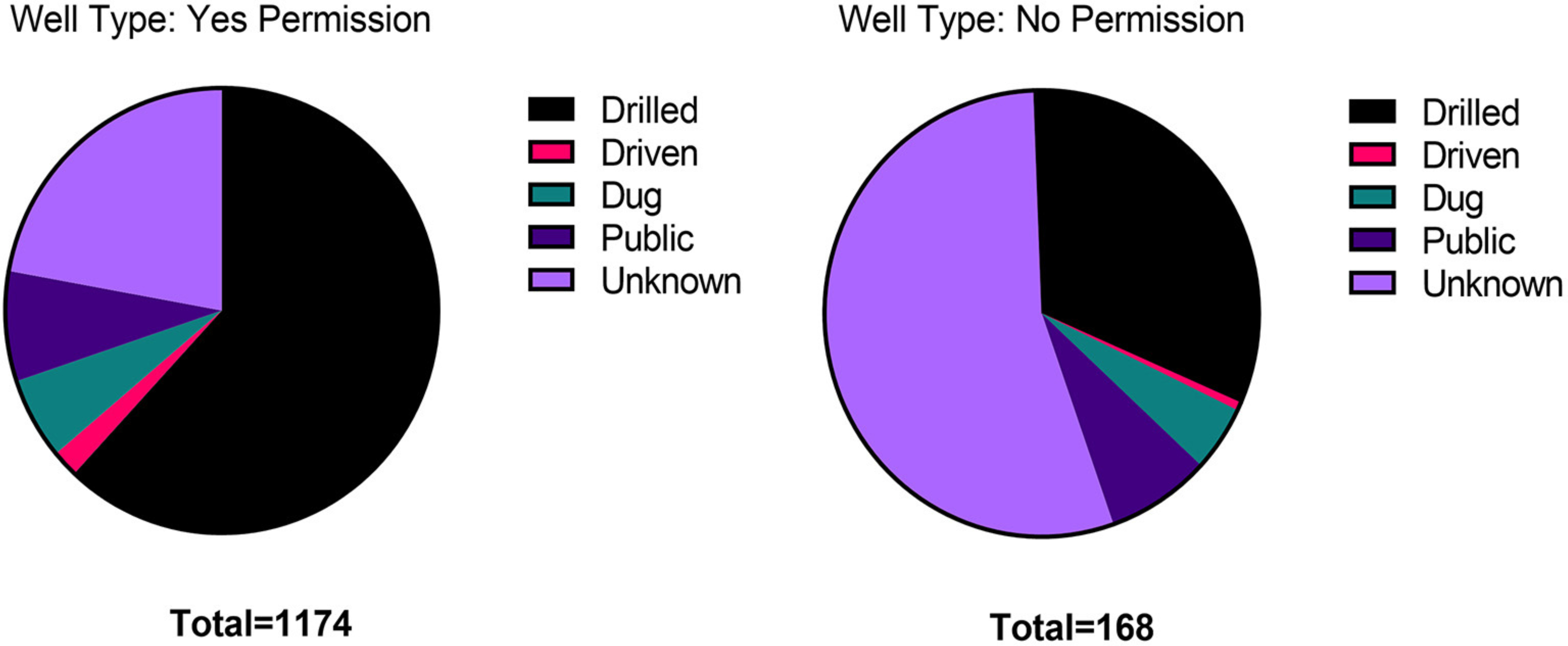
Permissions on Anecdata. A permission form was included in the datasheet for the “All About Arsenic” project, so that private data could be shared with state agencies charged with protecting public health. People who did not know much about their wells tended to not give permission for sharing any data.

In analyzing the data from those participants who gave permission to share their private data with the Maine CDC or New Hampshire DES, we noted that a higher percentage of people who did not provide permission to share their data did not know the source of their drinking water as compared to those who did provide permission to share their data (Figure 7). We are interested in pursuing the link between participant confidence in their data reporting and their willingness to have their private data shared. There may be information or features on Anecdata that could be provided to project participants that would increase their confidence in their data reporting and sharing.

Additional features that were developed for Anecdata resulted from addressing challenges related to the “All About Arsenic” project, such as ways to safely export data for use on other platforms like Tuva without disclosing information in privileged datasets. Though these features were created for the “All About Arsenic” project, all current and future projects have access to them as well.

### Power of Public Data

Data collected by citizen scientists have power to effect change when there is broad access to the data (Garbarino and Mason, 2016). Researchers can download the data and use it to guide their own research. In one example, a researcher at Maine Medical Center used the “All About Arsenic” dataset when they could not find the information that they needed in the Maine CDC’s Environmental Tracking Network dataset. In particular, the lead data in the well water dataset informed this researcher about the scope of lead problems across Maine and New Hampshire, and findings were incorporated into a grant application. In another example, staff from South Carolina Aquarium were able to use data collected as part of their “Litter-Free Digital Journal” project to testify to the city council in Folly Beach, South Carolina, leading to a ban on Styrofoam and reusable plastic bags.

### Future Directions

Anecdata is committed to offering features to ensure that citizen scientists have access to the data that they submit and that they can act on it when necessary. In an expansion of our thinking about “closing the citizen science data loop” (Disney et al., 2017), we plan to add improved data visualization, mapping, and communication features and a civic action toolkit to Anecdata to facilitate the use of data for improving public health, addressing issues like climate change, and informing public policy. Along these lines, we plan to add new types of spatial data collection (line and polygon) and new ways to interact with and map project data. Adding tools and new Web map functionality will allow project leaders and users to study the patterns of their project directly in the project without needing external software or accounts. We believe that this work is important, as maps can be strong communication tools especially for visual learners and communicators.

As we develop features for Anecdata, one of the key concerns is to ensure that their Agile development happens in a manner to support the privacy needs of **all stakeholders** involved in citizen science projects. The data access, visualization, and communication needs of the project administrators, the citizen science participants, and the general public need to be properly researched to ensure the **right balance of privacy features for individual stakeholders** across projects.

**FIGURE 8 |:**
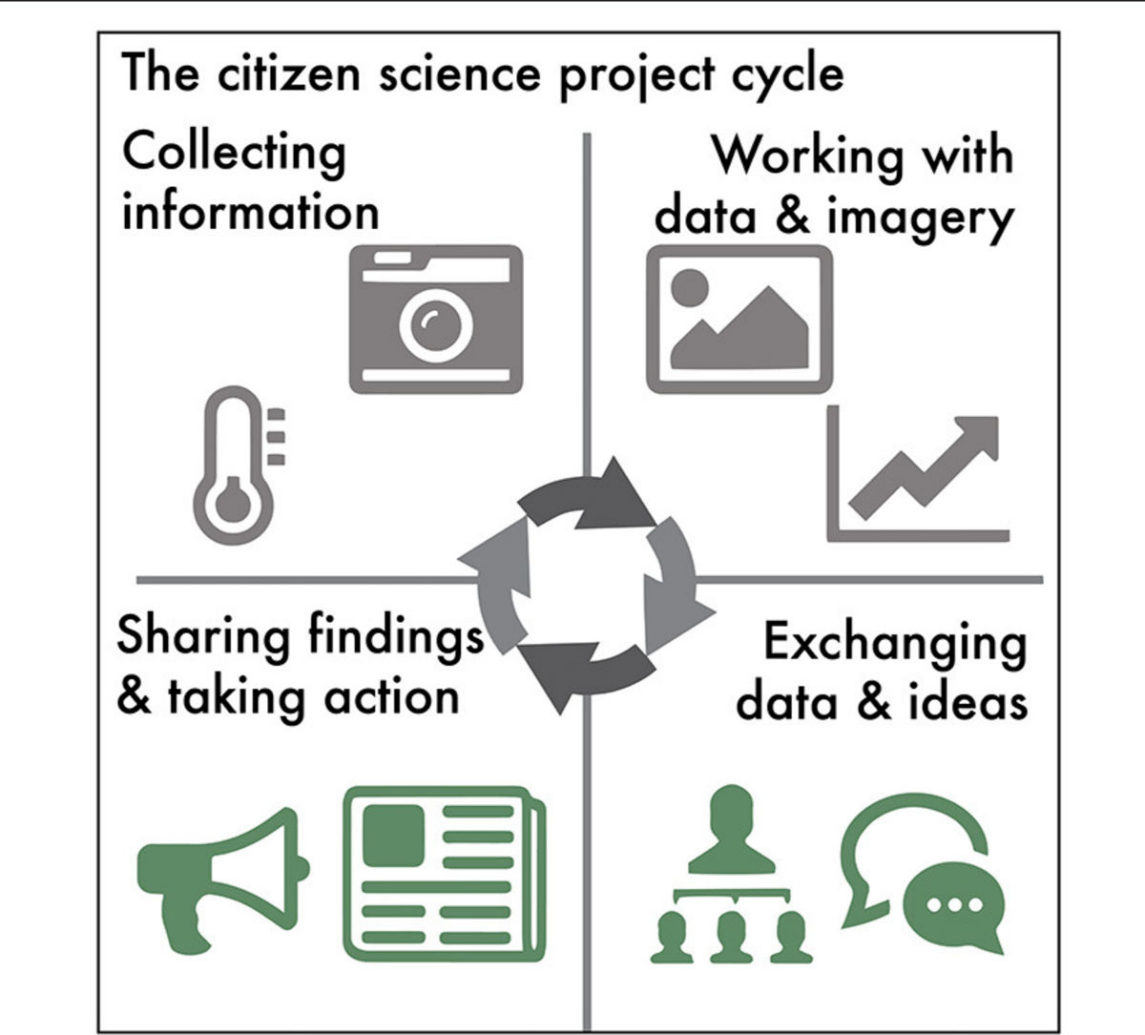
The citizen science project cycle. For citizen science data to have broad usage and applicability at various societal levels, technology platforms need to provide more than a way to set up projects for data collection. Project participants need a way to download and work with data and imagery to be able to tell data-supported stories that will lead to action.

Our vision is for Anecdata to provide the tools needed to assist users with engaging throughout the citizen science project cycle (Figure 8) not only in data collection and visualization but also in communicating with each other to make data-driven decisions and participate in civic action that leads to impactful and lasting change.

Although privacy is clearly an important feature for many projects, as evidenced by the rapid adoption of new privacy features by projects on Anecdata, project leaders should consider the extent to which data need to remain private. There will always be tension between data privacy and openness (Anhalt-Depies et al., 2019). The question emerges, what is the motivation for privacy of particular data types, and in what instances does it really confer any benefit to the parties involved, the place where data are being collected, or the species being documented. In the case of climate change, there is a lot at stake for the future of landscapes, habitats, and species. In trying to protect species by obscuring their location, for example, specific areas of concern (such as those impacted by flooding) may not be addressed. In these types of instances, the need for privacy must be balanced with the need for openness of data.

**FIGURE 9 |:**
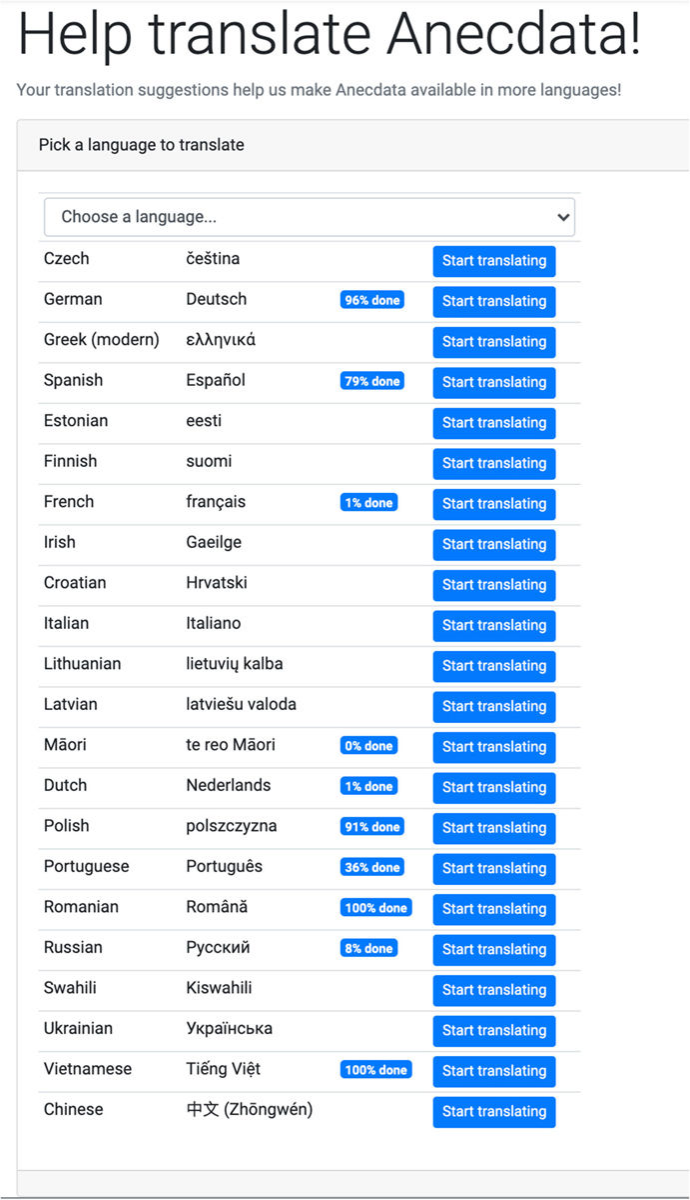
Translating Anecdata to promote findability, accessibility, interoperability, and reusability (FAIR) principles. Anecdata users are invited to help translate data into any of 22 languages.

Our collective experience with the development of privacy features has led us to explore ways to promote scientific data management and stewardship through adherence to principles of findability, accessibility, interoperability, and reusability (FAIR) (Wilkinson et al., 2016). Along these lines, we have facilitated collaborative efforts by the Anecdata community to provide translation of Anecdata into multiple languages to improve its **accessibility** across diverse geographic locations worldwide (Figure 9). Anecdata also has a “CSV Data uploader” feature available for project administrators that allows them to format and upload **legacy data** (from old datasets such as Excel sheets and databases) directly into Anecdata and make them **interoperable and reusable** with existing datasets. Anecdata provides APIs to researchers upon request that allow them to easily **access and reuse** anonymized datasets across projects.

Additional research and development efforts are currently underway to ensure that we enhance **findability (search), accessibility, interoperability, and reusability** of datasets across all projects on Anecdata, while ensuring that “private fields” and sensitive data (like personal information and geolocation) are only accessible to project administrators (or organizations) running citizen science projects on Anecdata.

Even though some projects may choose to make data open to the public for moving data to action, adequate information and support in terms of privacy, safety, and security of sensitive information must be provided to project administrators at regular intervals to further the Agile development of the Anecdata platform to meet privacy needs of various projects.

The development of privacy features for the “All About Arsenic” project set the stage for other projects to use privacy features across various local contexts and in support of different needs. Our journey into the development of privacy features showcases a genuine need for investment of time and effort into a usability study to help improve privacy features on Anecdata, which we plan to implement as a “next step” for Anecdata. We anticipate that continued development and refinement of key privacy features will be essential to supporting the diverse projects currently on Anecdata and those that will use Anecdata in the future. By providing an array of refined options for data privacy, Anecdata may be able to serve as a platform for a myriad of data collection projects that would benefit from but otherwise not be amenable to a citizen science approach.

## Figures and Tables

**TABLE 1 |:** Definitions of privacy features on Anecdata.org.

Privacy feature on Anecdata	Definition

Geoprivacy	The partial obscuring of geographic coordinates using an algorithm to make observations appear in the general area of the actual observation but shifted by a random distance to obscure the precise observation location.
Private fields	A feature that redacts certain datasheet questions from the publicly available dataset.
Anonymity	A feature that obscures the user account that was used to create an observation.

**TABLE 2 |:** Metadata in “All About Arsenic” project.

Personal information	Sample metadata

Associated school	Sample number
Name	Sample location
Street and mailing addresses	Sample filtration (Y/N)
Previous arsenic test	Type of filtration
Permission to share data	Water filtration description

**TABLE 3 |:** Anonymity conditions and user access.

Condition	Can the user edit the observation?

The user is an administrator in the observation’s project	Yes
The user created the observation (the observation’s **user_id** is the same as the logged-in user)	Yes
The user created the observation (There is a record in **anonymous_post_owners** with a **post_id** matching the observation’s ID and a **user_id** matching the logged-in user’s ID)	Yes
Default if no other condition is met	No

**TABLE 4 |:** Obfuscation algorithm.

<? php
function roundCoord($number = 0){
// Handle missing coordinates correctly:
if(empty($number)){
return 0;
}
// Generate a random floating-point
// number between−0.1 and 0.1
$randomNumber = (rand(0, 2000) - 1000) / 1000;
return $number + ($randomNumber / 10);
}

**TABLE 5 |:** Projects with privacy features on anecdata.

Project	Organization	Project location	#Of observations	#Of participants	Uses private features	Uses geoprivacy	Uses participant anonymity

Downeast Maine Smelt Monitoring	Downeast Salmon Federation	Eastern Maine	225	32	No	Yes	No
Eastern Meadowlark Survey	Mass Audubon - Bird Conservation Department and Mass Division of Fisheries & Wildlife	Massachusetts	992	117	No	Yes	No
All About Arsenic	MDI Biological Laboratory	Maine and New Hampshire	2,255	959	Yes	Yes	Yes
Coastal SOS Monitoring	Maine Signs of the Seasons	Maine	670	29	No	Yes	No
Beaver Survey	The Wetlands Conservancy	Oregon	383	110	No	Yes	No
MaMA Monitoring Plots Network	Ecological Research Institute	Eastern United States	668	158	Yes	No	No
Salamander Crossing Brigades	Harris Center for Conservation Education	New Hampshire	68	10	Yes	No	No
NASA’s Lower the Boom	NASA	United States	296	100	No	No	Yes
Terrapin Tracking Team	The Maritime Aquarium, CT DEEP, WCSU, CT DOT	Southwestern Connecticut	89	22	Yes	Yes	No
Great Green Crab Hunt	Kejimkujik National Park	New England	114	72	Yes	No	No
The Great Canadian Green Crab Hunt		Kejimkujik National Park Seaside, NS	24	8	Yes	No	No
VietFarm Network Update	VietFarm	Vietnam	58	56	Yes	No	No
Cover It Up: Using plants to control buckthorn	University of Minnesota, Department of Forest Resources	Minnesota	381	110	No	Yes	No
Barn & Cliff swallow nesting sites	MASS AUDUBON	Massachusetts	31	25	No	Yes	Yes
Copper River Steward’s Clean-up Journal	Copper River Watershed Project and Eyak Preservation Council	Alaska	16	7	No	Yes	No
Spidey Senser	University of Maryland, Baltimore County	United States	17	8	No	Yes	No
What is in my Backyard?	GreenDubs, University of Washington	Washington	335	87	No	Yes	No
Arsenic in All Seasons	College of the Atlantic	Mt. Desert Island, Maine	361	4	Yes	Yes	Yes
Dartmouth Dragonfly Mercury Project	Dartmouth College	Hanover, NH	715	7	No	No	Yes
Crowd the Tap Maine	Schoodic Institute at Acadia National Park	Winter Harbor, Maine	160	12	Yes	No	No
Rumex Hypogaeus around Christies creek		Christies Beach, South Australia	49	1	No	Yes	No
WildCam Vashon	Vashon Nature Center	Vashon Island, Washington	19	9	No	Yes	No
Salt Marsh Restoration and Citizen Science in Charleston, SC	South Carolina Sea Grant Consortium	Charleston, SC	36	32	Yes	Yes	No

## Data Availability

The datasets presented in this study can be found at https://www.anecdata.org.

## Footnotes

1
https://www.anecdata.org/projects/view/299

2
http://www.allaboutarsenic.org/

3
https://www.anecdata.org/projects/view/319

4
https://www.anecdata.org/projects/view/521

5
https://www.anecdata.org/projects/view/187

6
https://www.anecdata.org/projects/view/301

7
https://www.anecdata.org/projects/view/299

8
https://www.anecdata.org/projects/view/791

9
https://www.anecdata.org/projects/view/473

10
https://www.anecdata.org/projects/view/710
